# Automated Prediction of Osteoarthritis Level in Human Osteochondral Tissue Using Histopathological Images

**DOI:** 10.3390/bioengineering10070764

**Published:** 2023-06-25

**Authors:** Ateka Khader, Hiam Alquran

**Affiliations:** Department of Biomedical Systems and Informatics Engineering, Hijjawi Faculty for Engineering Technology, Yarmouk University, Irbid 21163, Jordan

**Keywords:** osteoarthritis, histopathological, hematoxylin eosin, safranin O fast green, DarkNet-19, MobileNet, NasNet, ResNet-101, ShuffleNet, PCA, ALO

## Abstract

Osteoarthritis (OA) is the most common arthritis and the leading cause of lower extremity disability in older adults. Understanding OA progression is important in the development of patient-specific therapeutic techniques at the early stage of OA rather than at the end stage. Histopathology scoring systems are usually used to evaluate OA progress and the mechanisms involved in the development of OA. This study aims to classify the histopathological images of cartilage specimens automatically, using artificial intelligence algorithms. Hematoxylin and eosin (HE)- and safranin O and fast green (SafO)-stained images of human cartilage specimens were divided into early, mild, moderate, and severe OA. Five pre-trained convolutional networks (DarkNet-19, MobileNet, ResNet-101, NasNet) were utilized to extract the twenty features from the last fully connected layers for both scenarios of SafO and HE. Principal component analysis (PCA) and ant lion optimization (ALO) were utilized to obtain the best-weighted features. The support vector machine classifier was trained and tested based on the selected descriptors to achieve the highest accuracies of 98.04% and 97.03% in HE and SafO, respectively. Using the ALO algorithm, the F1 scores were 0.97, 0.991, 1, and 1 for the HE images and 1, 0.991, 0.97, and 1 for the SafO images for the early, mild, moderate, and severe classes, respectively. This algorithm may be a useful tool for researchers to evaluate the histopathological images of OA without the need for experts in histopathology scoring systems or the need to train new experts. Incorporating automated deep features could help to improve the characterization and understanding of OA progression and development.

## 1. Introduction

Osteoarthritis (OA) is the leading cause of pain and disability in working-age adults and the elderly [1,2]. OA is not a process of mechanical wear and tear as previously thought; instead, it is a whole-organ disease that is driven by the disruption of the balance of cartilage homeostasis, inflammatory mediators, genetic factors, and innate immunity [3,4,5]. Joint destruction in the knee can be severe in OA patients and can lead to total knee replacement (TKR). A better understanding of the pattern and initiation of OA in the knee could help in the understanding of OA progression and influence the selection of therapies.

The histopathology of cartilage is usually used to evaluate the in situ state of the cartilage tissue. Microscopic histopathological grading of osteochondral tissue is usually used to evaluate OA development ex vivo. The most common OA grading systems are the Osteoarthritis Research Society International (OARSI) [6] and Histological-Histochemical Grading System (HHGS) scoring systems [7]. Although the HHGS score system is the most often used for the histological scoring of osteoarthritic cartilage, it is usually used to evaluate the more severe OA specimens [8]. OARSI is the best choice for mild or earlier phases of OA and for investigating the progression of OA. In general, a sensitive grading system that is able to detect early OA and its progression could be of great interest for drug development and OA research [9]. Moreover, the identification of early OA and the progression of OA is important in the development of early interferences and therapeutic techniques that could prevent the progression of OA [10].

Manual histopathological scoring systems can be time-consuming and need pathologists with years of experience and/or the training of new scorers [11]. Automatic OA evaluation and assessment based on histopathological image classification are very limited. Manual scoring systems are widely used for evaluation of the OA histopathological images. Machine learning and deep learning have aided massive data analyses, pattern identification, decision-making, and the production of accurate predictions [12]. Machine learning and deep learning were used for the histopathological grading of different tissues, using magnetic resonance imaging (MRI) [13,14], optical microscopy [15], and ultrasound [16].

The prediction and classification of the OA progression of the osteochondral tissue using machine learning and deep learning have been proposed in the literature; these methods were based on magnetic resonance imaging (MRI) [17,18] and radiography [19]. A deep convolutional neural network (CNN) was used to automatically diagnose hip OA using 420 hip X-ray images [20]. The results showed that the CNN model had 95% sensitivity and 92.8% accuracy as compared to the conventional manual assessment by physicians. In another study, deep learning was used for the automatic segmentation and subregional assessment of MRI images of articular cartilage and compared to manual segmentation [21]. Tiulpin et al. studied the use of deep learning and leveraged an ensemble of residual networks with 50 layers to predict OARSI and Kellgren–Lawrence (KL) grades of OA from knee radiographs [22]. The detection of the presence of OA using their model yielded an average precision of 0.98 and an area under the ROC curve (AUC) of 0.98.

However, few studies have looked at automation in the grading of histopathological samples. Rytky et al. used regularized linear and logistic regression models for the histopathological grading of osteochondral specimens imaged with contrast-enhanced microcomputed tomography (microCT) [23]. The models were trained against the manually graded histopathological samples to predict the grades of degeneration for the articular cartilage of the surface, deep, and calcified cartilage zone. They found that the model could detect the degeneration in the surface zone with an average precision of 0.89 (AUC of 0.92) while the detection of degeneration in the deep zone was the lowest, with an average precision of 0.46 (AUC of 0.62) [23]. Power et al. used supervised deep learning to automate the grading system for the histological images of engineering cartilage tissue [24]. Safranin O and fast green (SafO) was used for staining the engineered tissue; then, two experts graded the images. Transfer learning using a pre-trained DenseNet model was used to automate the scoring of the histological images; the scoring resulted in errors comparable to inter-user errors [24].

In this study, we aim to automate the classification of histopathological grading into early, mild, moderate, and severe OA using machine learning and deep learning techniques. The histological images of the osteochondral specimens were obtained from Venkata et al. [25]. The current methods could be improved with the development of methods for the analysis and grading of osteochondral histological samples, particularly as most researchers use manual grading for the histological samples. The developed methods could be used not only for the OA histological samples harvested after total knee arthroplasty but also for tissue engineering models of articular cartilage.

## 2. Materials and Methods

**Figure 1:**
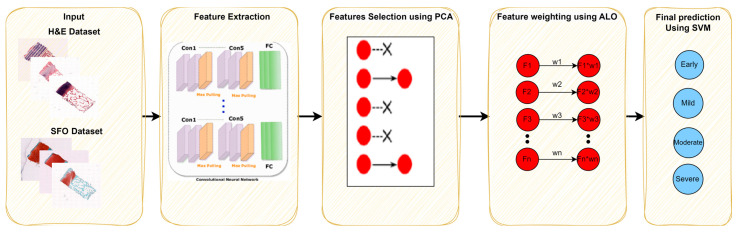
The proposed method for distinguishing the severity levels for both hematoxylin and eosin (HE) and safranin O and fast green (SafO) histopathological images.

The method proposed in this paper is shown in Figure 1; then, each block is explained in the following sections.

As is clear in Figure 1, the histopathological images passed through various stages: from deep learning structures, the extraction of feature maps, and the employing of PCA to the weighting optimization algorithm. The evaluation criteria are calculated in each stage. The corresponding sections clarify the novelty of the proposed approach.

### 2.1. Database

**Figure 2:**
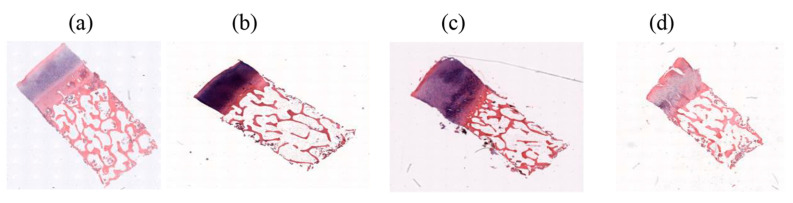
Representative HE-stained images of cartilage specimens, indicating (**a**) early, (**b**) mild, (**c**) moderate, and (**d**) severe OA.

**Figure 3:**
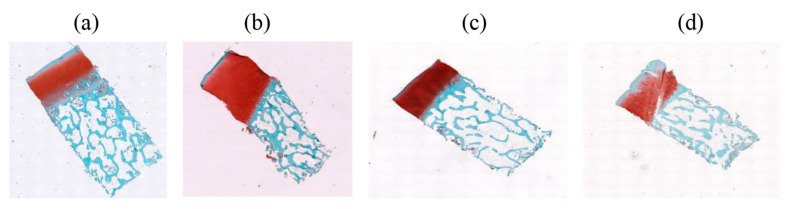
Representative SafO-stained images of cartilage specimens, indicating (**a**) early, (**b**) mild, (**c**) moderate, and (**d**) severe OA.

The osteochondral images were obtained from the database of Venkata et al. [25] (Available: https://doi.org/10.18735/77ye-yh24 (accessed on 2 February 2023)). Briefly, the samples were harvested from 90 patients undergoing total knee arthroplasty. Two osteochondral specimens (4 × 4 × 8 mm) were obtained, one from the medial (CM) and one from the lateral (CL), from the lateral femoral condyle. The specimens were stained with hematoxylin and eosin (H&E) or safranin O and fast green (SafO). SafO staining is usually used for staining glycosaminoglycans [26] and hematoxylin and eosin (H&E) staining is usually used for staining nuclei and extracellular proteins [27]. The samples were previously graded according to the OARSI grading system by three scorers 3 times (separated by at least 3 months) [25]. According to the average grades of the scorers, we divided the images of HE and SafO into early, mild, moderate, and severe OA, as shown in Figure 2 and Figure 3. In the OARSI scoring system, the score for early is less than 3.4, for mild it is 2.4–8.6, for moderate it is 8.6–15.4, and for severe it is 15.4–24 [28].

### 2.2. Deep Learning Features

Deep learning features represent the graphical descriptors for each class. They are inherent to the categories themselves. In this paper, several pre-trained deep learning models are employed to differentiate various levels of OA in two types of stained histological images (HE and SafO). The utilization of pre-trained convolutional neural networks (CNNs) to discriminate between two kinds of histological images does not provide accurate results. Therefore, the proposed method combines deep learning, machine learning, and optimization techniques to achieve high accuracy in predicting OA levels. The proposed method depends mainly on extracting the most representative features from the last fully connected model in each CNN. The deep learning structures were trained on the ImageNet database to classify 1000 classes. The transfer learning technique that was utilized to maintain the established structures is compatible with the desired problem statement, which focused on anticipating four levels of histological OA images. The transfer learning was made applicable by augmenting the input size of the image to make it suitable for the input layer of each one. Moreover, removing the last fully connected layer reduced it to four levels. The deep descriptors for each model were extracted from the last fully connected layer. Each one supplied four representative attributes for four levels for both types of stained images (HE and SafO) [29,30]. The utilized networks were ResNet-101, MobileNet, ShuffleNet, NasNet, and DarkNet-19. The idea behind using various structures is based on the ability of each one to extract features in a different manner and to learn in various ways, either in deep or in multiscale resolution. This leads to the obtaining of more representative features that can accurately represent the histopathological OA images.

#### 2.2.1. DarkNet-19

The DarkNet-19 is a type of CNN that consists of 19 convolutional layers, followed by a max-pooling layer and then two fully connected layers. DarkNet architecture is similar to that of VGGNet but with fewer parameters. It is applied to computer vision tasks such as object detection, image classification, and segmentation. Moreover, it was introduced as a part of YOLO (You Only Look Once), which is designed for tracking real-time objects [31].

#### 2.2.2. NasNet

NasNet stands for neural search architecture networks. This CNN is a well-known predefined convolutional neural network, which is trained over the ImageNet dataset with over 1000 classes from nature. The NasNet internal structure consists of a multi-series of cells. There are two types of cells: normal and reduction cells. The normal cells are responsible for extracting the graphical descriptors and producing the feature maps via convolutional filters. On the other hand, the reduction cell is in charge of reducing the size of the feature map’s width and height by a factor of 2. NasNet is ended by a SoftMax layer that allows obtaining the probability of classification task [31].

#### 2.2.3. ResNet-101

Residual neural networks are convolutional neural networks pre-trained over the ImageNet database; there are various versions based on the number of convolutional layers (Res-18,50, and 101). This kind of CNN is distinguished by its residual block property, which overcomes the vanishing gradient that appears due to deep learning. The skip connections lead to the bypassing of some of the neural layers and the feeding of the output of one layer as the input to the next level, which provides a different path for the gradient in backpropagation. That is the architecture of the residual block. ResNets consist of the stacking of such blocks. By transfer learning, the input image must be augmented to be compatible with ResNet input size 224 × 224 × 3, and the last fully connected layer must be replaced by another one that is suitable for the intended classification task [31,32].

#### 2.2.4. ShuffleNet

ShuffleNet is one of the most well-known pre-trained CNNs; it is appropriate for mobile applications. ShuffleNet executes two types of convolution to achieve a high level of accuracy. They are the point-wise convolution and the channel convolution; they lead to reduced time computation and make the results more accurate. The ShuffleNet structure consists of the stacking of shuffle netblocks; each one includes a point-wise convolutional layer and a depth-wise layer. The resultant output is passed to the ReLU layer for mapping purposes. The transfer learning is performed by augmenting the input data to be 224 × 224 × 3 and replacing the last fully connected layer to make it compatible with the number of intended classes [31].

#### 2.2.5. MobileNet

MobileNet is a pre-trained CNN designed for mobile and embedded devices. It is organized based on one depth-wise separable convolution that yields a reduction in the number of required parameters to maintain a good performance. The idea behind the depth-wise separable convolution is to split the convolution operation into two separate operations: a depth-wise convolution and a pointwise convolution. In a depth-wise convolution, each channel of the input is convolved with a separate filter, resulting in a set of feature maps. Then, a pointwise convolution is devoted to combining the attribute maps into the output by utilizing a 1 × 1 filter to convolve across all channels.

The MobileNet architecture consists of a series of convolutional layers, followed by global average pooling and a fully connected layer. The depth-wise separable convolution is performed in all these layers to obtain an efficient performance. The MobileNet structure may be adjusted by modifying the number of layers, filter sizes, and other hyperparameters [31,33].

### 2.3. Features Engineering

The features were extracted from each of the previously mentioned CNNs, four features for each CNN; the total number of extracted features from each type of stained image (HE or SafO) was 20 features. The extracted features underwent further processing techniques: through reduction by choosing the most significant or by weighting them using one of the most common optimization methods, which is known as the ant lion optimization technique.

#### 2.3.1. Principal Component Analysis

Principal component analysis (PCA) is well-known in data pre-processing and machine learning and is considered to be a feature selection algorithm. PCA transforms a high-dimensional dataset into a lower-dimensional space by identifying the principal components which explain the maximum variance in the datasets. PCA reduces the dimension of that dataset by preserving the most important information and discarding the redundant data task [29,30,31,32].

The principal components define the direction of the maximum variance in the extracted features. The following steps describe the process required to perform the PCA algorithm.

1. Standardization: this step is performed by standardizing each column feature that makes the mean for each feature zero, and the variance is unity.
2. Covariance matrix: this step is performed by constructing the covariance matrix, which is a square matrix that reflects the variance between each pair of features; its diagonal represents the variance for each feature and the off-diagonal represents the covariance between each pair of features.
3. Computation of the principal components: this step is performed by computing the eigenvector, which explains the direction of maxim variance, and the eigenvalue that quantifies the amount of maximum variance.
4. Selection of the principal components: the principal components are selected based on 95% of the majority variance of the features.
5. Mapping between the selected principal components and the features: this is performed by projecting the standardized features onto the best principal components.

#### 2.3.2. Feature Weighting Using ALO

Feature weighting represents the features that are more important than others when optimizing the classification problem; it reveals the role of each feature in the classification pattern by distinguishing by weight. The linear weight is proposed for the feature space to obtain a specific weight for the features; then, the new feature represents the original feature multiplied by its weight, as shown in the following equation:

(1) $$New_{Feature}=Weight\times Old\_Feature$$

Ant lion optimization (ALO) is a metaheuristic optimization algorithm that is used for tuning the parameters to achieve high accuracy. In this paper, we explored feature weights and the optimal value of k in the k-nearest neighbors (k-NN) algorithm; simultaneously, we used the accuracy of k-NN as a fitness function. The difference between PCA and ALO is that the former reveals the significant features and discards the less influential features. All the selected attributes have the same weight, which leads to an equal impact on the classification results. On the other hand, in this paper, the cascading of these two optimization techniques was the key to improving and obtaining the highest accuracies. The selected features were passed to the ALO algorithm to achieve an optimized weight for each one that was significant.

The ALO algorithm can be updated to search for a combination of feature weights and k values that optimize the performance of the k-NN model. The approach is performed using the accuracy of k-NN as a fitness function [34].

The steps of ALO are as follows:

1. Initialize the population of ant lions randomly.
2. Evaluate the accuracy of each ant lion in the population based on both weight and k-value.
3. Define the king ant lion based on the highest accuracy.
4. Move the ant lions towards the king ant lion using a certain formula that simulates the hunting behavior of the ant lions.
5. Calculate the accuracy for the new position.
6. Repeat steps 3–5 until the stopping criterion is met.
7. The results are the optimized weights.

### 2.4. Support Vector Machine

Support vector machines (SVMs) are popular supervised machine learning algorithms used in medical diagnosis. SVM is superior for both linear and non-linear separable data. SVM is used in the medical diagnosis field for discriminating between various classes, such as cancer, diabetics heart arrhythmia, cervical cancer, brain tumors, liver cancer, corneal ulcer, etc.

It is based on finding the optimal margin region for different classes and mapping the features to higher dimensional space using kernels to make the data separable in higher dimensional space. The kernel choice function has a significant impact on the performance of the classifier, in addition to the choosing of the relevant features. SVM is a powerful tool for medical diagnosis, and it is applied for different applications due to its reliability and high performance [35,36]. In this paper, we employed deep learning, feature engineering, and an SVM machine learning classifier to predict OA levels in human osteochondral tissue using histopathological images. The novel combination between them leads to build a reasonable system that can infer significant deep features and can weight them to obtain a reliable scoring diagnosis.

## 3. Results

The two types of stained images were passed to five pre-trained CNN models. The classification procedure was performed in four scenarios. First, deep learning classification was used to classify the four levels of OA. Second, deep learning features were extracted for each CNN and a support vector machine classifier was used to distinguish between the four levels for each type of stained image. Third, feature engineering techniques were applied to evaluate the most significant features from five CNNs using PCA. The last scenario reveals the importance of the feature weighting method by applying the ALO algorithm to give weight to each selected feature. The following subsections are devoted to discussing the obtained results in each scenario. The evaluation criteria that were used in this paper are those in [37].

(2) $$accuracy=\frac{TP+TN}{TP+TN+FP+FN}$$

(3) $$Recall=\frac{TP}{TP+FN}$$

(4) $$Precision=\frac{TP}{TP+FP}$$

(5) $$Specificity=\frac{TN}{TN+FP}$$

(6) $$F1-score=\frac{2\times Precision\times Recall}{Precision+Recall}$$

### 3.1. Pre-Trained Model Classification

Table 1 represents the accuracy for both the HE and the SafO images using DarkNet-19, MobileNet, NasNet, ResNet-101, and ShuffleNet. As is clear from Table 1, the accuracy of utilizing deep learning for HE images does not exceed 70.6% using NasNet. Moreover, the sensitivity and precision are too low, which leads to the F1 score being too low. Therefore, the deep convolution networks could not distinguish between various types of severity levels. For the SafO images, the accuracy ranged between 73.3% and 80% for the different CNN classifiers, among which DarkNet-19 had the highest accuracy. The obtained results were not promising; therefore, a hybrid model is recommended to extract the deep features and then pass them to a machine learning classifier to outperform the classification results.

### 3.2. Deep Features with SVM

**Figure 4:**
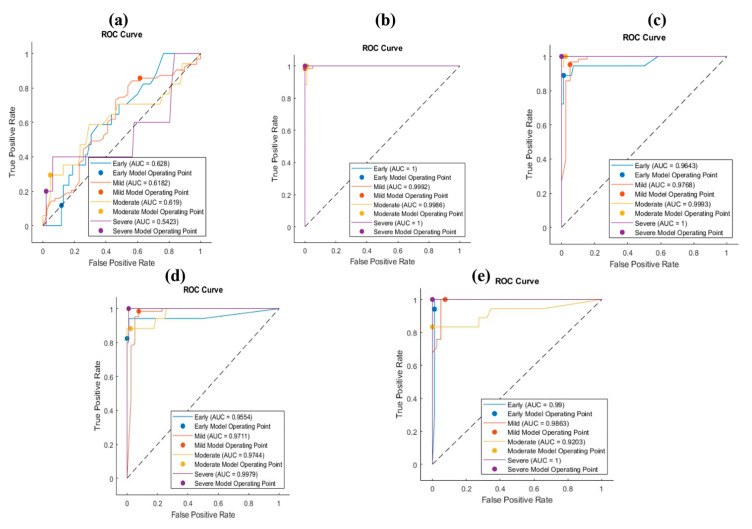
ROC curves of HE images for (**a**) deep DarkNet-19 features with SVM, (**b**) deep DarkNet-19 features with SVM, (**c**) deep DarkNet-19 features with SVM (**d**) deep DarkNet-19 features with SVM, and (**e**) deep DarkNet-19 features with SVM.

Four features were extracted from the last fully connected layer for each CNN. The deep features were passed to the SVM classifier. Table 2 and Table 3 show the performance of the classification for the HE images; the performance was enhanced except in the case of DarkNet-19. The enhancement comes from employing deep learning features and machine learning classifiers. The reason behind the worst performance of DarkNet-19 was the failure of DarkNet to extract the representative features for the four classes. The improved accuracy was 96% for the ShuffleNet features with the 3rd polynomial SVM classifier. The recall was the highest for the MobileNet features for the early class level. Moreover, the precision was also the best in MobileNet. The highest precision that was obtained was 100% for the severe class in MobileNet, NasNet, and ShuffleNet. On top of that, Figure 4 illustrates the receiver operating curve for each classification procedure. Each figure represents the relation between the true positive and the false positive rates. As the area under the curve (AUC) increases, the classifier has a high performance in distinguishing the particular classes. All the suggested CNNs had the AUC in all the classes, except DarkNet, which failed to extract the representative features for each class.

The same procedure was applied for the SafO images; the performances of each classifier with SVM are shown in Table 2 and Table 3. The performance of the DarkNet was much better than in the HE cases. The accuracy for all the CNN features with SVM ranged from 94.1% to 98% for ResNet-101 and MobileNet, respectively. The worst sensitivity was obtained for the ResNet-101 features for the moderate class. Nevertheless, the recall was almost high in all the classes for each network descriptor. The lowest positive predictive value for all the classes was greater than 85%. This indicates the ability of the extracted features to help in differentiating between various levels of severity.

**Figure 5:**
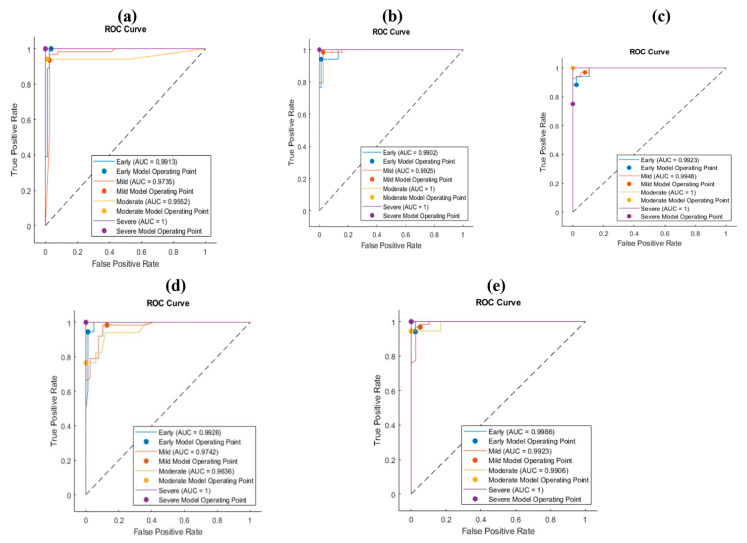
ROC curves of SaFO images for (**a**) deep DarkNet-19 features with SVM, (**b**) deep DarkNet-19 features with SVM, (**c**) deep DarkNet-19 features with SVM (**d**) deep DarkNet-19 features with SVM, and (**e**) deep DarkNet-19 features with SVM.

Moreover, for more analysis and clarification, the ROC curve (Figure 5) explains the impact of applying a hybrid process between deep learning and machine learning. The improvement of the AUC for each class, early, mild, moderate, and severe, reflects the ability of the proposed procedure to determine the kind of severity level for osteochondral tissue using SafO-stained images of human cartilage specimens, which imply cartilage structure, cell glycosaminoglycan content, and tide-mark integrity for the four types of severity levels, as we mentioned before: early, mild, moderate, and severe OA. To improve the performance of the proposed procedure using feature engineering techniques, the simplest method is to combine all the features from all the CNNs and then pass them to the kernel SVM to improve the results. The huge dimensions of using twenty features may lead to an increase in the computation time cost, which leads to the use of the principal component analysis (PCA). PCA is one of the most familiar methods for feature reduction that indicate up to 95% variance of the features. The proposed approach is to mix the benefits from all the CNNs and then find the significant features. The next section describes the results for PCA.

### 3.3. Principal Component Analysis (PCA)

All the features from the previous CNNs were fused and utilized to classify the images; then, PCA was devoted to the prediction of the most significant features. The twenty features from five CNNs were further processed under PCA to find the most significant subset features. Then, the most significant features passed to the SVM. The best obtained ten features for the HE images were:

1. Four features from MobileNet.
2. Three features from ShuffleNet.
3. Two features from NasNet.
4. One feature from ResNet-101.

**Figure 6:**
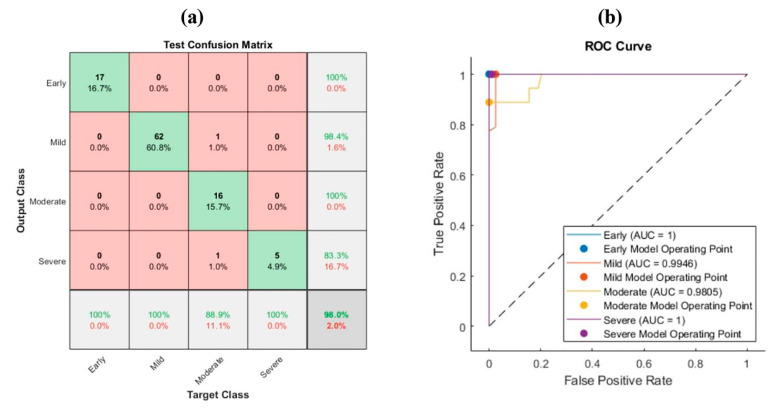
Feature fusion for HE images with PCA: (**a**) confusion matrix and (**b**) ROC curve.

**Figure 7:**
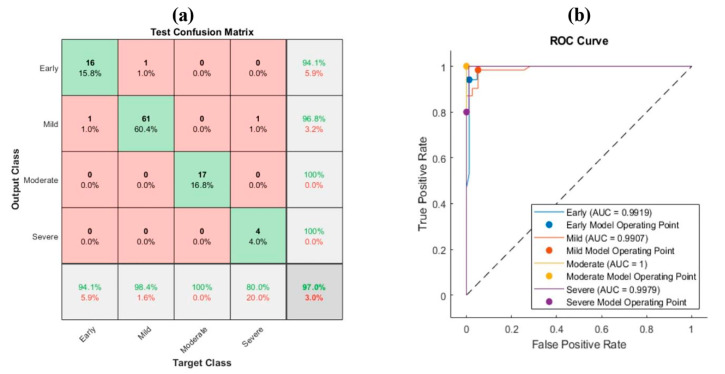
Feature fusion for SafO images with PCA: (**a**) confusion matrix and (**b**) ROC curve.

The most significant features did not involve any features from the DarkNet which was expected since the accuracy was low for the DarkNet. Figure 6 and Figure 7 show the confusion matrix of the PCA of all the features from all the convolution neural networks and the corresponding ROC curve for the HE and SafO images, respectively. Figure 6 describes the resultant confusion matrix and its corresponding ROC curve for the HE images. The accuracy was 98% for all the classes. On the other hand, the sensitivity for all the categories was 100%, except for the moderate level, which was 89%. However, the precision was 100% for the early and moderate levels, whereas it was 98.4% and 83.3% for the mild and severe levels, respectively. The AUC was 1 for the early and severe classes. On the other hand, the AUC was 0.995 for the mild class and 0.981 for the severe class. The obtained features using MobileNet performed better than those using the ten features. Therefore, after applying PCA for all the fused features, the most significant were the MobileNet features. They improved the previous results obtained using MobileNet features only.

The same procedure was applied to the fused features that were extracted from the SafO-stained images. The most significant features with 95% variance were ordered as follows:

1. Three features from MobileNet.
2. Three features from ShuffleNet.
3. Two features from NasNet
4. Two features from DarkNet

The ordering of the significant features satisfied the obtained results that employed features from each CNN individually. The highest accuracy appeared in MobileNet, then ShuffleNet. The worst accuracy was obtained using the ResNet-101 features. Therefore, they were not counted as significant features. Figure 7 describes the obtained results for the SafO-stained images using the most significant ten features.

The obtained accuracy was 97%. The highest recall was in the moderate category, whereas the lowest sensitivity was in the severe class. On top of that, the best precision was maintained in the moderate and severe classes. The lowest positive predictive value was in the early class. The area under the curve for all the classes was almost 1.

### 3.4. Ant Lion Optimization (ALO)

**Figure 8:**
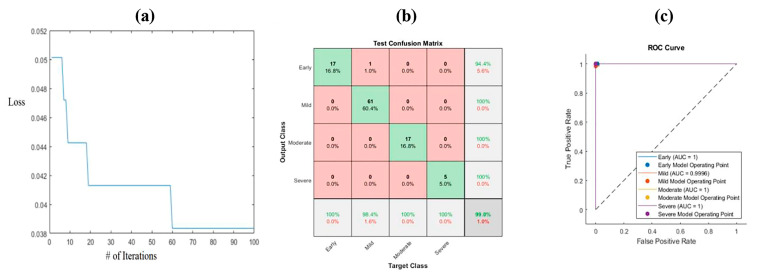
ALO algorithm for HE images: (**a**) convergence of the algorithm, (**b**) confusion matrix, and (**c**) ROC curve. Where # represents the number.

The ant lion optimization method combines the weights for each feature alongside the objective function, which is the loss of the convergence. The iterative procedure is performed to achieve the plateau of loss. This leads to the best weights for the features. The range of weights for each feature is [0–1]. The algorithm was applied to both kinds of images for all the extracted deep features. Figure 8 shows the convergence loss function versus the number of iterations for the HE images. As is clear from the figure, the maximum iteration is 100, and the convergence is constant after 60 iterations. The corresponding equation shows the optimized weight for each feature.

(7) $$\begin{array}{ll} y=0.522642\times F1 & +0.503514\times F2+0.093848\times F3+0.482934\times F4+0.11463\times F5+0.167205\times F6\\ & +0.750722\times F7+0.770949\times F8+0.159337\times F9+0.364798\times F10 \end{array}$$

where y represents the label of the image, and F1–F10 are the ten most significant features.

The confusion matrix of the obtained results is described in Figure 8b. The weighting features enhanced the accuracy to 99%. The sensitivity and precision were almost 100% for all the classes, except that the recall was 98.8% for the mild level and 94.4% for the early class. The ROC curve is illustrated in Figure 8c. The area under the curve was 1 for all the classes. The F1 score values were 0.97, 0.991, 1, and 1 for the early, mild, moderate, and severe classes, respectively (Table 4). The specificity values were 98.8%, 100%, 100%, and 100% for the early mild, moderate, and severe classes, respectively. As is clear from Table 4 and Figure 8, ALO has a higher performance than PCA in all the classes.

**Figure 9:**
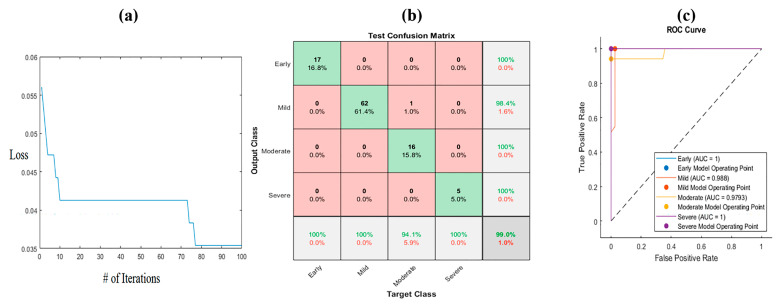
ALO algorithm for SafO images: (**a**) convergence of the algorithm, (**b**) confusion matrix, and (**c**) ROC curve. Where # represents the number.

The same procedure was applied for the SafO images; Figure 9a shows the number of iterations for the ALO algorithm versus the loss function. After 80 iterations, the loss function was constant, and the optimized weighted features were maintained. The optimized weights were:

(8) $$\begin{array}{ll} y=0.216401\times F1 & +0.898295\times F2+0.92736\times F3+0.110877\times F4\\ & +0.416086\times F5+0.749176\times F6+0.386958\times F7\\ & +0.67024\times F8+0.030166\times F9+0.584659\times F10 \end{array}$$

The achieved accuracy in the SafO images was the same as in the HE images (99%). The highest sensitivity was 100% in the early, mild, and severe categories. However, the highest precision was in the early, moderate, and severe levels. Figure 9c describes the AUC for the weighted features and the SVM classifier. The AUC was 1 in both the early and the severe classes, while the AUC was 0.979 in the moderate class and 0.988 in the mild class. The specificity was computed for all the levels, as follows: 100%, 97.4%, 100%, and 100% for the early, mild, moderate, and severe classes, respectively (Table 5). Furthermore, the F1 score values were 1, 0.971, 1, and 0.889 for the early, mild, moderate, and severe categories, respectively, using the PCA classifier, while the F1 score values were 1, 0.991, 0.97, and 1 for the early, mild, moderate, and severe categories, respectively, using the ALO classifier. As with the HE images, the ALO classifier performed better compared with PCA for the SafO images.

## 4. Discussion

In this study, we showed that machine learning and deep learning can be used to automatically classify the osteochondral histopathological images into early, mild, moderate, and severe OA. The manual histopathological scoring systems are time-consuming and need a trained scorer to grade the images. This study used five CNN models, including ResNet-101, MobileNet, ShuffleNet, NasNet, and DarkNet-19, to extract features from HE and SafO histopathological images of different levels of OA. As deep learning was insufficient to classify the OA images, we employed the deep features with a machine learning classifier to enhance the classification results, and we then optimized these features using various engineering methods, such as PCA and ALO. Although the deep learning method was first used in this manuscript to predict the severity of OA, the histopathological OA images were very complex due to the many changes that happen in both the cartilage and the subchondral bone during OA progression, such as the network of collagen fibers, the subchondral bone structure, the proliferation of chondrocytes, the size of cartilage change, and the proteoglycans loss, which results in surface cracking [38]. All of these make it very difficult for deep learning procedures alone to classify histopathological OA images. So, in this study, combinations of multiple algorithms were used with machine learning classifiers and various engineering methods, such as PCA and ALO. Combinations of different feature engineering approaches have been utilized in different studies due to the complexity of the images, the tissue, the type of images, and the sizes [39,40,41,42].

The results showed that the F1 score values were 0.97, 0.991, 1, and 1 for the early, mild, moderate, and severe classes, respectively, for the HE-stained images using the ALO classifier. For the SafO images, the F1 score values were 1, 0.991, 0.97, and 1 for the early, mild, moderate, and severe categories, respectively, using the ALO classifier. This study had a limitation in the dataset in that there was a very small number of images for the severe class. Only 14 images were available for the HE staining and another 14 images for the SafO staining for the severe class. So, we focused on reporting the F1 score since the data were imbalanced [43].

Few studies have utilized artificial intelligence to score or classify osteochondral or cartilage histopathological images. In another study, a machine learning technique was used to automatically grade 3D histopathological images of osteochondral samples to predict the degeneration of surface, deep, and calcified cartilage zones [23]. The samples were imaged using defect contrast-enhanced microCT. Transfer learning using a pre-trained ResNet-34 encoder was used. The model was able to predict the degeneration in the surface zone (AUC of 0.92 and AP of 0.89), followed by the calcified zone (AUC of 0.71 and AP of 0.65) and the deep zone (AUC of 0.62 and AP of 0.46) [23]. In another study, a deep learning technique was used to automate the grading of the histological images of engineered cartilage, in which the grading was classified into four categories [24]. Transfer learning using a pre-trained DenseNet model was used for feature extraction to automatically score the histological images of engineered cartilage. It was found that the RMSEs for the model prediction were in a similar range as the inter-user of 0.71 [24]. In our study, using the ALO algorithm for HE images, the specificity values were 98.8%, 100%, 100%, and 100% for early mild, moderate, and severe classes, respectively, and the AUC was 1 for all the classes. Using the ALO algorithm for the SafO images, the specificity values were 100%, 97.4%, 100%, and 100% for the early, mild, moderate, and severe classes, respectively, and the AUC values were 1, 0.988, 0.979, and 1 for the early, mild, moderate, and severe classes, respectively.

Machine and deep learning have recently been used to investigate OA development and progression using MRI or X-ray images [44,45,46,47]. Ashinsky et al. used machine learning to investigate the development of OA using the MRI images of 68 patients. A hierarchy of algorithms representing morphology (WND-CHRM) was used to classify the development of OA with 75% accuracy [17]. In another study, the T2 relaxation time of the MRI images of the 4384 subjects with and without OA was analyzed using DenseNet and random forests to distinguish OA [45]. The DenseNet training model attained a sensitivity equal to 74.53% and a specificity equal to 76.13%, which was comparable to the random forest model with a sensitivity of 67.01% and a specificity of 71.79%. Tolpadi et al. used a DenseNet CNN to predict the total knee replacement (TKR) from the MRI images and the clinical and demographic information of patients with OA and patients without OA [48]. Their model was able to predict the TKR with the AUCs of 0.834 ± 0.036 and 0.943 ± 0.057 for patients with OA and without OA, respectively.

In OA, the integrity of collagen and glycosaminoglycan, which give the cartilage the mechanical properties, is compromised [49]. The articular cartilage has a complex structure without blood vessels or nerves, making it difficult to repair or to treat the cartilage defect. So, the progression of OA has been investigated by many researchers using a manual grading system [25,50,51]. Saarakkala et al. studied the collagen and proteoglycan changes during OA progression using the OARSI histopathology grading system [52]. Then, a composition-based finite element (FE) model was employed to study the tissue function. Mantripragada et al. investigated the scoring of polarized light microscopy (PLM) images as a potential method to understand early OA as compared with the standard histopathological methods [50]. They found that adding a PLM scoring system helped in the characterization of early and mild OA. OA progression and development have also been studied in many animal models of human OA [53,54,55]. A whole joint microCT image scoring and histologic scoring systems of a Hartley guinea pig, which is considered a model of human OA, were investigated to determine the changes in articular cartilage and bone [55]. The grading was conducted by two experts using the OARSI guidelines. So, automating the grading system of histopathological methods could help in understanding OA progression and development.

## 5. Conclusions

The proposed methods revealed the ability of the integration between deep learning, machine learning, and feature engineering in scoring the severity levels of OA. The deep learning models help the researcher in the classification and extraction of the representative features of each category. The feature engineering method enhanced the performance of the classification results, which focused on obtaining the most important attribute in addition to giving them a specific weight. The best results obtained in this study were obtained by using PCA followed by ALO then SVM classifiers. To the best of our knowledge, this is the first study that handles the combination between PCA and ALO to obtain the best classification. Moreover, this is the first study that discusses the employment of artificial intelligence in OA microscopic histopathological images. In this study, we were able to build an artificial intelligence model that could distinguish the different stages of the OA from the osteochondral histopathological images without the need of human experts, which could be of great interest to the researchers and scientific community. Furthermore, the model could be modified for the evaluation of tissue engineering cartilage formation instead of using the manual grading system.

## Figures and Tables

**Table 1:** The accuracy using different CNN structures for HE and SafO images.

	CNN	DarkNet-19	MobileNet	NasNet	ResNet-101	ShuffleNet
Images						
HE		69.6%	61.8%	70.6%	69.6%	64.7%
SafO		80.2%	77.2%	73.3%	76.2%	74.3%

**Table 2:** The accuracy using different CNN structures with SVM classifier for HE and SafO images.

	CNN	DarkNet-19	MobileNet	NasNet	ResNet-101	ShuffleNet
Images						
HE		60.8%	99%	95.1%	94.1%	96.1%
SafO		95%	98%	95%	94.1%	95%

**Table 3:** The precision and sensitivity using different CNN features with SVM classifier for HE and SafO images.

		HE Images		SafO Images	
Class		Sensitivity	Precision	Sensitivity	Precision
Early	DarkNet-19	11.8%	16.7%	100%	85.7%
	MobileNet	100%	100%	94.1%	94.1%
	NasNet	89.9%	94.1%	88.2%	88.2%
	ResNet-101	82.4%	100%	94.4%	94.4%
	ShuffleNet	94.1%	94.1%	94.1%	88.9%
Mild	DarkNet-19	85.7%	69.2%	93.5%	98.3%
	MobileNet	98.4%	100%	98.4%	98.4%
	NasNet	95.2%	96.8%	96.8%	95.3%
	ResNet-101	98.4%	95.4%	98.4%	92.4%
	ShuffleNet	100%	95.4%	96.8%	96.8%
Moderate	DarkNet-19	29.4%	55.6%	94.1%	94.1%
	MobileNet	100%	94.4%	100%	100%
	NasNet	100%	98.5%	100%	100%
	ResNet-101	88.2%	88.2%	76.5%	100%
	ShuffleNet	83.3%	100%	94.4%	100%
Severe	DarkNet-19	20%	16.7%	100%	100%
	MobileNet	100%	100%	100%	100%
	NasNet	100%	100%	75%	100%
	ResNet-101	100%	83.3%	100%	100%
	ShuffleNet	100%	100%	100%	100%

**Table 4:** The performance of feature engineering on HE-stained images.

Class	Feature Engineering	Sensitivity	Precision	Specificity	F1 Score
Early	PCA	100%	100%	100%	1
	ALO	100%	98.4%	98.8%	0.97
Mild	PCA	100%	98.4%	97.5%	0.991
	ALO	100%	98.4%	97.5%	0.991
Moderate	PCA	88.9%	100%	100%	0.941
	ALO	100%	100%	100%	1
Severe	PCA	100%	83.3%	99%	0.909
	ALO	100%	100%	100%	1

**Table 5:** The impact of feature engineering on SafO images.

Class	Feature Engineering	Sensitivity	Precision	Specificity	F1 Score
Early	PCA	94.1%	94.1%	98.8%	1
	ALO	100%	100%	100%	1
Mild	PCA	98.4%	96.8%	94.8%	0.971
	ALO	100%	98.4%	97.4%	0.991
Moderate	PCA	100%	100%	100%	1
	ALO	94.1%	100%	100%	0.97
Severe	PCA	80%	100%	100%	0.889
	ALO	100%	100%	100%	1

## Data Availability

The data presented in this study are available upon request from the corresponding author.
